# *Notes from the Field*: Intimate Partner Homicide Among Women — United States, 2018–2021

**DOI:** 10.15585/mmwr.mm7334a4

**Published:** 2024-08-29

**Authors:** Adam Rowh, Shane Jack

**Affiliations:** ^1^Epidemic Intelligence Service, CDC; ^2^Division of Violence Prevention, National Center for Injury Prevention and Control, CDC.

**Summary**

**What is already known about this topic?**

Stressors associated with the COVID-19 pandemic prompted concern about a possible increase in intimate partner violence.

**What is added by this report?**

The overall rate of intimate partner homicide among women remained stable from 2018–2019 to 2020–2021. However, during 2020–2021, the proportion of victims who were non-Hispanic Black or African American women increased, and suspects were more frequently previously known to law enforcement.

**What are the implications for public health practice?**

The exacerbation of racial disparities highlights the importance of comprehensive prevention efforts and further research into the influences of structural factors on intimate partner violence, including homicide. Contact with law enforcement represents a potential missed opportunity for preventing future violence.

Stay-at-home orders and other stressors associated with the COVID-19 pandemic prompted concerns about a possible increase in intimate partner violence (*1*), including intimate partner homicide, which disproportionately affects women (*2*). Subsequent research on this topic has produced inconsistent results (*3*). CDC analyzed changes in the incidence and characteristics of intimate partner homicide during January 1, 2018–December 31, 2021, using data from the National Violent Death Reporting System (NVDRS) (*4*).

## Investigation and Outcomes

### Data Source and Analysis

This report summarizes NVDRS data covering 49 states (all except Florida), the District of Columbia, and Puerto Rico. Analysis subjects included female victims of intimate partner homicide* aged ≥18 years. Population crude rates (female intimate partner homicide deaths per 100,000 women) were calculated for 2018–2019 and 2020–2021.^†^ Selected characteristics of the victim, suspected perpetrator (suspect), and incident were tabulated. Observations were compared between periods using Wilcoxon rank sum and Pearson’s chi-square tests as appropriate; p-values <0.05 were considered statistically significant. This activity was reviewed by CDC, deemed not research, and was conducted consistent with applicable federal law and CDC policy.^§^

### Overall Intimate Partner Homicide Characteristics

During 2018–2021, a total of 3,991 female victims of intimate partner homicide were reported to NVDRS (Table). The median victim age was 38 years; 49.3% were non-Hispanic White (White), 29.9% were non-Hispanic Black or African American, (Black), 14.8% were Hispanic or Latino (Hispanic), and 6.0% comprised all other races and ethnicities.^¶^ Incidents most often occurred at the victim’s residence (68.0%) and involved a male suspect (98.5%), a single victim (61.4%), and a firearm (66.6%). In addition, 20.3% of the suspects were known to have a previous history of abusing the victim, 15.8% had suspected alcohol or substance use near the time of the incident, 14.7% had previous contact with law enforcement during the 12 months preceding the homicide, and 6.0% were known to have mental illness that directly contributed to the homicide** (*4*).

**TABLE:** Number, percentage,* and rate^†^ of intimate partner homicides^§^ among females aged ≥18 years, by victim, suspect, and incident characteristics — National Violent Death Reporting System, United States,^¶^ 2018–2021

Characteristic	No. (%)			
	Overall N = 3,991	2018–2019 n = 1,832	2020–2021 n = 2,159	p-value**
**Overall rate (homicides per 100,000 women)**	**0.96**	**0.97**	**0.95**	**0.39**
**Victim characteristics**				
**Age, yrs, median (IQR)**	**38 (29–50)**	39 (29–50)	38 (29–50)	0.06
**Race and ethnicity**				
Black or African American, non-Hispanic	**1,193 (29.9)**	501 (27.3)	692 (32.1)	<0.01
White, non-Hispanic	**1,968 (49.3)**	938 (51.2)	1,030 (47.7)	0.03
Hispanic or Latino	**590 (14.8)**	272 (14.8)	318 (14.7)	0.93
All others^††^	**240 (6.0)**	121 (6.6)	119 (5.5)	0.16
**Suspect characteristics**				
Suspect age, yrs, median (IQR)	**41 (31, 54)**	41 (32, 54)	40 (30, 53)	0.15
Male sex	**3,908 (98.5)**	1,797 (98.7)	2,111 (98.4)	0.48
History of abusing the victim	**811 (20.3)**	355 (19.4)	456 (21.1)	0.17
Homicide was direct result of suspect’s mental illness^§§^	**238 (6.0)**	114 (6.2)	124 (5.7)	0.52
Suspected alcohol or substance use in hours preceding incident	**631 (15.8)**	303 (16.5)	328 (15.2)	0.25
Previous contact with law enforcement (past 12 mos)	**587 (14.7)**	230 (12.6)	357 (16.5)	<0.01
**Incident characteristics**				
**Incident type**				
Single-victim homicide	**2,451 (61.4)**	1,087 (59.3)	1,364 (63.2)	0.01
Single-victim homicide followed by suspect suicide	**1,151 (28.8)**	557 (30.4)	594 (27.5)	0.05
Multivictim homicide	**191 (4.8)**	83 (4.5)	108 (5.0)	0.50
Multivictim homicide followed by suspect suicide	**153 (3.8)**	80 (4.4)	73 (3.4)	0.12
All others**^¶¶^**	**45 (1.1)**	25 (1.4)	20 (0.9)	0.23
Selected precipitating circumstances***				
Argument preceding incident	**1,700 (42.6)**	755 (41.2)	945 (43.8)	0.10
Injured at victim's residence	**2,714 (68.0)**	1,263 (68.9)	1,451 (67.2)	0.24
Jealousy or “love triangle”	**307 (7.7)**	165 (9.0)	142 (6.6)	<0.01
**Method of injury**				
Firearm	**2,660 (66.6)**	1,193 (65.1)	1,467 (67.9)	0.06
Sharp instrument	**598 (15.0)**	294 (16.0)	304 (14.1)	0.09
Personal and strangulation	**390 (9.8)**	188 (10.3)	202 (9.4)	0.34
Blunt instrument	**193 (4.8)**	81 (4.4)	112 (5.2)	0.27
All others^†††^	**150 (3.8)**	76 (4.1)	74 (3.4)	0.24

* Percentages might not sum to 100% because of rounding.

^†^ Rates (intimate partner homicides per 100,000 women) were calculated using population data from CDC WONDER. Denominators for the rates for California, Illinois, Pennsylvania, and Texas represent the population of the counties from which the data were collected.

^§^ Incidents defined as intimate partner homicides reflected the following relationships between victim and suspected perpetrator: spouse, ex-spouse, girlfriend or boyfriend, ex-girlfriend or ex-boyfriend, and girlfriend or boyfriend with unknown current relationship status.

^¶^ Forty-five states reported statewide data. California, Illinois, Pennsylvania, and Texas reported data from selected counties representing a subset of their population during this period. Data for Florida were excluded because the data did not meet the completeness threshold for circumstances.

** Wilcoxon rank sum test was performed for continuous variables; Pearson's chi-square test was performed for categorical variables. P-values <0.05 were considered statistically significant.

^††^ Victims who were not Hispanic or Latino, Black or African American, or White were combined into a heterogenous group to avoid low count suppression. This group consisted of the following proportions of persons: 47.5% Asian or Pacific Islander, 30.0% American Indian or Alaska Native, 16.7% multiracial (two or more races or ethnicities), 5.4% other or unspecified race or ethnicity, and 0.8% of unknown race or ethnicity.

^§§^ Including alcohol problem, current depressed mood, current diagnosed mental health problem, current mental health or substance use treatment, history of ever being treated for mental health or substance use problem, other addiction, or other substance use problem. Complete variable definition available at https://pubmed.ncbi.nlm.nih.gov/37220104/.

**^¶¶^** Includes multiple homicides followed by legal intervention deaths, mutual homicide/shootout, and other unclassified multiple deaths.

*** Denominator includes those homicides with one or more precipitating circumstances (3,984). The sums of percentages in columns exceed 100% because more than one circumstance could have been present per victim.

^†††^ Includes poisoning, falls, explosives, drowning, fire or burns, shaking, motor vehicles including buses and motorcycles, other transport vehicles, intentional neglect, biological weapons, or other unclassified weapons.

### Comparison of Intimate Partner Homicide Rates During 2018–2019 and 2020–2021

The rates of intimate partner homicide during 2018–2019 (0.97 per 100,000) and 2020–2021 (0.95) were not significantly different (p = 0.39). During the two periods, most incident characteristics were similar, including the proportion of victims injured at their residence (2018–2019 = 68.9%; 2020–2021 = 67.2%; p = 0.24). However, during 2020–2021, victims were more frequently Black (32.1%, versus 27.3% during 2018–2019; p<0.01) and less frequently White (47.7%, versus 51.2% during 2018–2019; p = 0.03), and suspects more frequently had previous law enforcement contact (16.5%, versus 12.6% during 2018–2019; p<0.01). During 2020–2021, homicides more frequently involved a single victim (63.2%, versus 59.3% during 2018–2019; p = 0.01).

## Preliminary Conclusions and Analysis

Overall rates and most characteristics of intimate partner homicide involving female victims in the United States did not significantly change during 2018–2021. Black women were disproportionately victims of intimate partner homicide throughout the study period (i.e., during this period, Black women constituted approximately 13.4% of the population but accounted for 29.9% of intimate partner homicide victims); this disparity widened during 2020–2021. Further, during 2020–2021, the proportion of suspects in intimate partner homicide incidents who had contact with law enforcement during the preceding 12 months increased approximately 30%, suggesting a potential missed opportunity for prevention. These findings highlight the importance of a comprehensive approach to violence prevention such as that summarized in CDC’s Prevention Resources for Action (https://www.cdc.gov/violence-prevention/php/resources-for-action). Future research is needed on the role that structural factors play in the risk for intimate partner homicide, including those related to risk for violence in general (*5*).

## Footnotes

* Incidents defined as intimate partner homicides reflected the following relationships between victim and suspected perpetrator: spouse, ex-spouse, girlfriend or boyfriend, ex-girlfriend or ex-boyfriend, and girlfriend or boyfriend with unknown current relationship status.

† Denominators for the rates for California, Illinois, Pennsylvania, and Texas represent the population of the counties from which the data were collected. https://wonder.cdc.gov/single-race-v2021.html

§ 45 C.F.R. part 46, 21 C.F.R. part 56; 42 U.S.C. Sect. 241(d); 5 U.S.C. Sect. 552a; 44 U.S.C. Sect. 3501 et seq.

¶ Victims who were not Black, White, or Hispanic were combined into a heterogenous group to avoid low count suppression. This group consisted of persons who were Asian or Pacific Islander (47.5%), American Indian or Alaska Native (30.0%), multiracial (16.7%), other or unspecified race or ethnicity (5.4%), and unknown race or ethnicity (0.8%).

** Including alcohol problem, current depressed mood, current diagnosed mental health problem, current mental health or substance use treatment, history of ever being treated for mental health or substance use problem, other addiction, or other substance use problem.

## References

[1] Evans ML, Lindauer M, Farrell ME. A pandemic within a pandemic—intimate partner violence during covid-19. N Engl J Med 2020;383:2302–4. 10.1056/NEJMp2024046 32937063

[2] Smith E. Just the stats: female murder victims and victim-offender relationship, 2021. Washington, DC: US Department of Justice, Bureau of Justice Statistics; 2022. https://bjs.ojp.gov/female-murder-victims-and-victim-offender-relationship-2021.

[3] McNeil A, Hicks L, Yalcinoz-Ucan B, Browne DT. Prevalence & correlates of intimate partner violence during COVID-19: a rapid review. J Fam Violence 2023;38:241–61. 10.1007/s10896-022-00386-6 35368512 PMC8961087

[4] Nguyen BL, Lyons BH, Forsberg K, Surveillance for violent deaths—National Violent Death Reporting System, 48 states, the District of Columbia, and Puerto Rico, 2021. MMWR Surveill Summ 2024;73(No. SS-5):1–44. 10.15585/mmwr.ss7305a1 38980822 PMC11262823

[5] Armstead TL, Wilkins N, Nation M. Structural and social determinants of inequities in violence risk: a review of indicators. J Community Psychol 2021;49:878–906. 10.1002/jcop.22232 31421656 PMC7278040

